# Infant Gut Microbiota Development Is Driven by Transition to Family Foods Independent of Maternal Obesity

**DOI:** 10.1128/mSphere.00069-15

**Published:** 2016-02-10

**Authors:** Martin Frederik Laursen, Louise B. B. Andersen, Kim F. Michaelsen, Christian Mølgaard, Ellen Trolle, Martin Iain Bahl, Tine Rask Licht

**Affiliations:** a National Food Institute, Technical University of Denmark, Søborg, Denmark; b Department of Nutrition, Exercise and Sports, University of Copenhagen, Frederiksberg, Denmark; University of Wisconsin, Madison

**Keywords:** 16S rRNA sequencing, breastfeeding, complementary diet, family foods, infant gut microbiota, maternal obesity

## Abstract

The first years of life are paramount in establishing our endogenous gut microbiota, which is strongly affected by diet and has repeatedly been linked with obesity. However, very few studies have addressed the influence of maternal obesity on infant gut microbiota, which may occur either through vertically transmitted microbes or through the dietary habits of the family. Additionally, very little is known about the effect of diet during the complementary feeding period, which is potentially important for gut microbiota development. Here, the gut microbiotas of two different cohorts of infants, born either of a random sample of healthy mothers (*n =* 114), or of obese mothers (*n =* 113), were profiled by 16S rRNA amplicon sequencing. Gut microbiota data were compared to breastfeeding patterns and detailed individual dietary recordings to assess effects of the complementary diet. We found that maternal obesity did not influence microbial diversity or specific taxon abundances during the complementary feeding period. Across cohorts, breastfeeding duration and composition of the complementary diet were found to be the major determinants of gut microbiota development. In both cohorts, gut microbial composition and alpha diversity were thus strongly affected by introduction of family foods with high protein and fiber contents. Specifically, intake of meats, cheeses, and Danish rye bread, rich in protein and fiber, were associated with increased alpha diversity. Our results reveal that the transition from early infant feeding to family foods is a major determinant for gut microbiota development.

**IMPORTANCE** The potential influence of maternal obesity on infant gut microbiota may occur either through vertically transmitted microbes or through the dietary habits of the family. Recent studies have suggested that the heritability of obesity may partly be caused by the transmission of “obesogenic” gut microbes. However, the findings presented here suggest that maternal obesity *per se* does not affect the overall composition of the gut microbiota and its development after introduction of complementary foods. Rather, progression in complementary feeding is found to be the major determinant for gut microbiota establishment. Expanding our understanding of the influence of complementary diet on the development and establishment of the gut microbiota will provide us with the knowledge to tailor a beneficial progression of our intestinal microbial community.

## Abstract (precis)

The potential influence of maternal obesity on infant gut microbiota may occur either through vertically transmitted microbes or through the dietary habits of the family. Recent studies have suggested that the heritability of obesity may partly be caused by the transmission of “obesogenic” gut microbes. However, the findings presented here suggest that maternal obesity *per se* does not affect the overall composition of the gut microbiota and its development after introduction of complementary foods. Rather, progression in complementary feeding is found to be the major determinant for gut microbiota establishment. Expanding our understanding of the influence of complementary diet on the development and establishment of the gut microbiota will provide us with the knowledge to tailor a beneficial progression of our intestinal microbial community.

## INTRODUCTION

Despite the temporal resilience and stability of the gut microbiota, long-term diet (1) and major diet shifts (2) are known to affect the human gut microbiota. Infancy and early childhood constitute a period in life in which the microbiota is characterized by relatively low stability and high responsiveness toward influencing factors. During this period, dietary factors have major implications for the establishment of the gut microbiota (3, 4). While many previous studies have focused on early infant diet (5, 6), particularly breastfeeding and formula feeding, only a few have addressed the effects of the complementary (solid-food) diet of infants in the period after 6 months of age (7). As the gut microbial population is not fully established until the age of 3 to 5 years (8, 9), it is important to understand how it is influenced by the transition from early infant feeding to family foods during the complementary feeding period, which is defined by the WHO as the period from 6 until 18 to 24 months of age (10). The established adult gut microbiota has been linked with a range of metabolic, autoimmune, and allergic diseases (9). Specifically, the intestinal microbiome has repeatedly been linked to obesity in animal models (11–15) as well as in human studies (16, 17). By transplantation of fecal microbial communities from human twins discordant for obesity into germfree mice, it has been shown that a greater increase in body mass and adiposity occurs in mice transplanted with obese donor microbiota than with the corresponding microbiota from the lean donor twin, suggesting the importance of gut microbes over human genetics in the etiology of obesity (18). Indeed, children of obese parents have a higher risk of developing obesity, and this is not explained solely by human genetic predisposition. Since gut microbes can be transferred from mother to infant during birth (19), an obesity-associated microbiota may be transferred from an obese pregnant woman to her offspring. It is well documented that obese mothers on average breastfeed for a shorter time than normal-weight mothers (20) and that breastfeeding has a protective effect on obesity in offspring (21). Further, parental obesity is associated with lower socio-economic status and specific dietary patterns (22) that may affect the type of complementary diet introduced to the infant and thereby the development of the gut microbiota (9), as well as contribute to future obesity risk (23). Indeed, diet-microbiota interactions have been shown to be key players in the development of obesity (18). Therefore, we compared the gut microbiota profiles of two different cohorts of Danish infants at the ages of 9 and 18 months, designated SKOT I (24) and SKOT II (25), respectively (SKOT is a Danish abbreviation for dietary habits and well-being of young children). SKOT I includes infants from a random sample of mothers (mean body mass index [BMI], 22.9 kg/m^2^), and SKOT II includes infants of obese mothers (mean BMI, 35.1 kg/m^2^). To elucidate the impact of (i) maternal obesity and (ii) dietary factors on infant gut microbiota development, associations between specific features of the gut microbiota and dietary factors were investigated with a focus on breastfeeding and complementary diet composition.

## RESULTS

### Gut microbiota development during the complementary feeding period is independent of maternal obesity.

**FIG 1:**
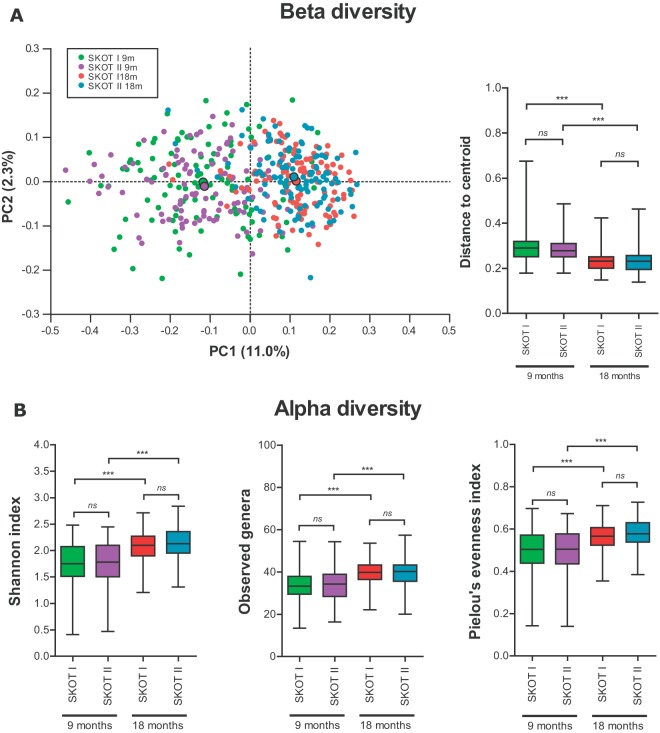
Gut microbial beta and alpha diversity is independent of maternal obesity but changes over time. (A) PCoA plot based on Bray-Curtis dissimilarity, with the centroid for each group shown with a black boarder. The distance to the group centroid for each point provides a measure of homogeneity of variance, used to estimate beta diversity. PC1 and PC2, principal coordinates 1 and 2, respectively. (B) Alpha diversity measures as estimated by the Shannon index, observed genera, and Pielou’s evenness index. Boxes indicate 25th to 75th percentiles, with mean values marked as a line and whiskers indicating minimum and maximum values. ns, not significant; *, *P* < 0.05; **, *P* < 0.01; ***, *P* < 0.001 (according to Tukey’s honestly significant difference test for beta diversity and paired [within cohorts, across time points] or unpaired [across cohorts at the same time point] *t* tests for alpha diversity measures).

**FIG 2:**
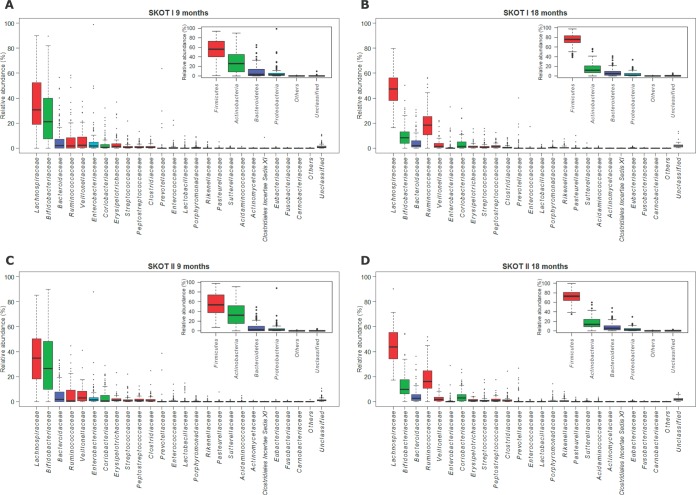
Composition of gut microbiota in SKOT I and SKOT II. Relative abundances of bacterial phyla (small panels) and families (large panels) in the SKOT I cohort (*n =* 114) at the ages of 9 months (A) and 18 months (B) and in the SKOT II cohort (*n =* 113) at the ages of 9 months (C) and 18 months (D). Boxes indicate 25th to 75th percentiles, with mean relative abundances marked as lines and whiskers indicating the range (minimum/maximum) multiplied by the interquartile range (25th to 75th percentiles) from the boxes. Bacterial families are ranked by average relative abundances at the age of 9 months. Detailed information can be found in Table S1 in the supplemental material.

**FIG 3:**
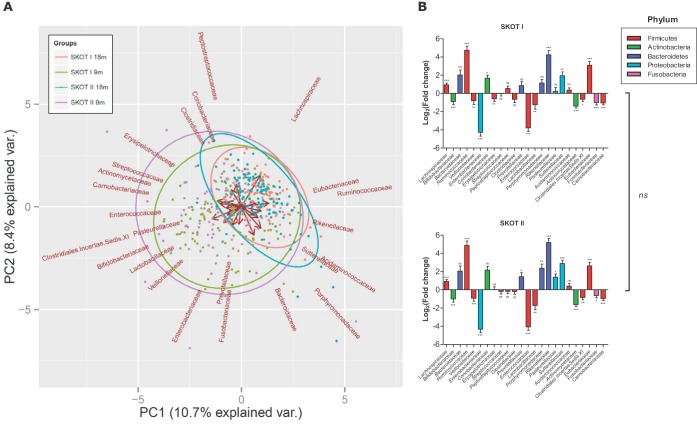
Gut microbiota composition is independent of maternal obesity but changes over time. (A) PCA biplot of the relative abundances of bacterial families at 9 and 18 months of age in SKOT I and SKOT II. Ellipses indicate 95% confidence intervals for each group, while arrows show loadings. var., variance. (B) Log_2_-transformed fold changes of relative abundances of bacterial families between the ages of 9 and 18 months within SKOT I and SKOT II. Error bars indicate the standard error of the mean. ns, not significant; *, *q* < 0.05; **, *q* < 0.01; ***, *q* < 0.001 (according to false-discovery-rate-corrected [5%] paired Wilcoxon signed-rank tests of relative abundances at 9 months versus 18 months). No significant differences were found between the fold changes of bacterial families occurring in the two cohorts after we performed false-discovery-rate-corrected (5%) Mann-Whitney tests.

To assess the impact of maternal obesity on gut microbiota establishment in offspring, we sequenced the V3 region of 16S rRNA genes from fecal samples of 227 individuals at both 9 and 18 months of age in the two SKOT cohorts. These cohorts are different with respect to maternal obesity and generally differ in terms of socio-economic status, C-section prevalence, and early infant feeding but differ only slightly with respect to infant body composition measures (Table 1). Between-sample diversity (beta diversity) of the gut microbiota in the two cohorts was investigated by principal-coordinate analysis (PCoA) of the Bray-Curtis dissimilarity indices and showed clustering according to age rather than cohort (Fig. 1A). Distances to the group centroid for each point, as an estimate of beta diversity, illustrated no differences between cohorts (Fig. 1A). However, greater beta diversity was observed at 9 months than at 18 months in both cohorts, in line with previous reports (26–28). Levels of within-sample diversity (alpha diversity), as estimated by the Shannon index, the number of observed genera, and Pielou’s evenness index of the communities, were not significantly different between the two cohorts at either 9 months or 18 months of age (Fig. 1B). However, there was a significant increase in these alpha diversity measures from 9 to 18 months in both cohorts. On a compositional level, the gut microbiotas across time and cohorts were dominated by four phyla, *Firmicutes* (64.2%), *Actinobacteria* (23.4%), *Bacteroidetes* (7.7%), and *Proteobacteria* (4.3%), while less than 0.5% belonged to other phyla or were unclassified. On average, 98.3% of the communities belonged to 24 bacterial families (Fig. 2). Despite large interindividual variation, average bacterial communities assessed at the phylum level as well as at the family level at 9 months and 18 months were highly similar between SKOT I and II (Fig. 2). Indeed, according to PCA of family-level composition, samples clustered according to age rather than cohort and showed the relative contributions of bacterial families to the variation in the data set (Fig. 3A). After correction for multiple testing, we found no significant differences between cohorts with respect to relative abundances of bacterial phyla, families, or genera at either 9 or 18 months, and no differences in the changes occurring from 9 to 18 months between the two cohorts were identified (Fig. 3B). In contrast, over time, *Lachnospiraceae*, *Ruminococcaceae*, *Eubacteriaceae*, *Rikenellaceae*, and *Sutterellaceae* were significantly increased in both cohorts, and *Bifidobacteriaceae*, *Actinomycetaceae*, *Veillonellaceae*, *Enterobacteriaceae*, *Lactobacillaceae*, *Enterococcaceae*, *Clostridiales incertae* *sedis* XI, *Carnobacteriaceae*, and *Fusobacteriaceae* were significantly decreased in both cohorts (Fig. 3B; see Table S1 in the supplemental material). This is in agreement with a previous study of the SKOT I cohort using quantitative-PCR (qPCR)-based microbiota assessment (8) and with studies involving other cohorts (27–30). These results suggest that maternal obesity *per se* does not influence gut microbiota development during the complementary feeding period. The high gut microbiota similarity between the two cohorts, independently sampled during different time periods, allowed a high-powered characterization of infant gut microbiota development and identification of the main factors explaining variation in gut microbiota.

10.1128/mSphere.00069-15.3 Table S1  Bacterial families/genera that are significantly different in relative abundance between 9 and 18 months in either SKOT I or SKOT II or both. Download Table S1, DOCX file, 0.03 MB. Copyright © 2016 Laursen et al. 2016 Laursen et al. This content is distributed under the terms of the Creative Commons Attribution 4.0 International license.

**TABLE 1:** Characteristics of the SKOT cohort subsets used in this study^a^

Parameter (unit of measure)	Value for SKOT I (*n* = 114)	Value for SKOT II (*n* = 113)	*P* value^b^
Mother			
BMI at the infant age of 9 mo (mean kg/m^2^ ± SD)	22.9 ± 3.2	35.1 ± 4.2	<0.0001 (MWT)
		
Work situation			
Job (%)	80.7	76.1	
Student (%)	14.9	8.0	
No job (%)	4.4	15.9	0.007 (χ^2^)
		
Education level			
Basic (%)	12.3	32.7	
Short (%)	11.4	12.4	
Medium (%)	32.5	34.5	
Long (%)	43.9	20.4	<0.0001 (χ^2^)
		
Household income per year^c^			
<650,000 DKK (%)	44.7	49.0	
≥650,000 DKK (%)	55.3	51.0	0.587 (FET)
			
Infant			
Birth			
Wt for age at birth (mean *Z* score ± SD)	0.35 ± 0.84	0.81 ± 1.04	0.0003 (*t*W)
Length for age at birth (mean *Z* score ± SD)	1.20 ± 0.96	1.65 ± 1.11	0.013 (*t*)
BMI for age at birth (mean *Z* score ± SD)	−0.41 ± 0.91	−0.08 ± 1.13	0.015 (*t*W)
C-section prevalence (%)	13.3	33.7	0.0006 (FET)
Gestational age at birth (mean no. of wks ± SD)	40.2 ± 1.2	40.3 ± 1.3	0.448 (*t*)
		
Sex			
Male (%)	47.4	53.1	
Female (%)	52.6	46.9	0.427 (FET)
		
Early infant feeding			
Age at introduction to complementary feeding (mean no. of mos ± SD)	4.4 ± 0.7	4.2 ± 0.6	0.0018 (MWT)
Duration of exclusive breastfeeding (mean no. of mos ± SD)	3.6 ± 1.8	2.6 ± 2.0	0.0006 (MWT)
Total duration of breastfeeding (mean no. of mos ± SD)	8.1 ± 3.8	6.6 ± 4.5	0.0068 (MWT)
		
Anthropometry			
Wt for age at 9 mo (mean *Z* score ± SD)	0.46 ± 0.92	0.83 ± 0.93	0.003 (*t*)
Length for age at 9 mo (mean *Z* score ± SD)	0.23 ± 0.90	0.88 ± 0.97	<0.0001 (*t*)
BMI for age at 9 mo (mean *Z* score ± SD)	0.45 ± 1.03	0.46 ± 0.95	0.939 (*t*)
Subscapularis skinfold thickness for age at 9 mo (mean *Z* score ± SD)	0.19 ± 1.24	0.39 ± 0.99	0.184 (*t*W)
Waist circumference at 9 mo (mean cm ± SD)	45.64 ± 3.07	44.95 ± 2.99	0.090 (*t*)
Wt for age at 18 mo (mean *Z* score ± SD)	0.50 ± 0.84	0.70 ± 0.85	0.094 (*t*)
Length for age at 18 mo (mean *Z* score ± SD)	0.07 ± 0.92	0.42 ± 0.97	0.006 (*t*)
BMI for age at 18 mo (mean *Z* score ± SD)	0.64 ± 0.97	0.63 ± 0.87	0.945 (*t*)
Subscapularis skinfold thickness for age at 18 mo (mean *Z* score ± SD)	0.59 ± 1.11	0.82 ± 1.20	0.141 (*t*)
Waist circumference at 18 mo (mean cm ± SD)	46.83 ± 2.88	46.61 ± 2.52	0.550 (*t*)

a Data are from reference 25.

b Statistical significance was evaluated by the Mann-Whitney test (MWT), the chi-square test (χ^2^), Fischer’s exact test (FET), Student’s *t* test (*t*), and Student’s *t* test with Welch’s correction (*t*W).

c DKK, Danish krone.

### Limited influence of C section, gestational age at birth, and prior use of antibiotics.

The mode of delivery (31), gestational age at birth (within a normal range of full-term delivery) (32), and use of antibiotics (33) have all previously been shown to impact the infant gut microbiota. In the SKOT cohorts, neither microbial community compositions nor alpha diversity measures at 9 months were significantly different between individuals born by C section and those born vaginally (see Table S2 in the supplemental material). We did, however, note a decreased relative abundance of *Bacteroidaceae* (*P* = 0.003, false-discovery-rate-corrected *P* values [*q*] = 0.072) in infants born by C section in SKOT II (Table S2), in line with results of previous studies (27, 31, 34). Gestational age at birth was not associated with gut microbiota composition or alpha diversity at 9 months (Table S3), and the use of antibiotics 2 weeks before the sample was taken (current antibiotic use was an exclusion criterion) could not explain the variation in gut microbial diversity at 9 or 18 months (Table S4). All infants in the present study were delivered at full term (range, 37 to 42 weeks), C-section prevalence was low in SKOT I (Table 1), and for only a few infants was the use of oral antibiotics during the 2 weeks prior to sampling registered (10 individuals in total). Further, the relative late sampling point (9 months of age) may explain discrepancies with prior studies.

10.1128/mSphere.00069-15.4 Table S2  Gut microbial alpha diversity and composition at 9 months of age in infants born by C-section versus those born vaginally. Download Table S2, DOCX file, 0.02 MB. Copyright © 2016 Laursen et al. 2016 Laursen et al. This content is distributed under the terms of the Creative Commons Attribution 4.0 International license.

10.1128/mSphere.00069-15.5 Table S3  Average gestational age at birth and correlations to gut microbial alpha diversity and composition at the age of 9 months. Download Table S3, DOCX file, 0.02 MB. Copyright © 2016 Laursen et al. 2016 Laursen et al. This content is distributed under the terms of the Creative Commons Attribution 4.0 International license.

10.1128/mSphere.00069-15.6 Table S4  Gut microbial alpha diversity of infants with or without 14 days’ prior use of oral antibiotics at the ages of 9 and 18 months. Download Table S4, DOCX file, 0.01 MB. Copyright © 2016 Laursen et al. 2016 Laursen et al. This content is distributed under the terms of the Creative Commons Attribution 4.0 International license.

### Duration of exclusive breastfeeding, rather than age at introduction of complementary feeding, is reflected in late-infancy gut microbiota.

**FIG 4:**
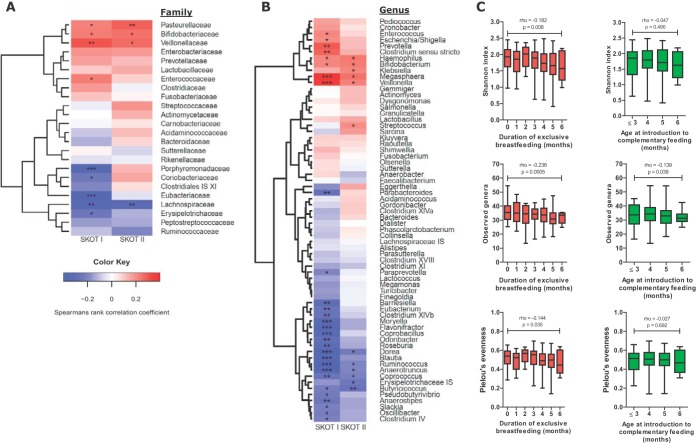
Duration of exclusive breastfeeding is reflected in late-infancy gut microbiota. Hierarchical clustering of Spearman’s rank correlations of duration of exclusive breastfeeding with gut microbial composition at 9 months of age at the family (A) and genus (B) levels in SKOT I and II. *, *P* < 0.05; **, *P* < 0.01; ***, *P* < 0.001. (C) Spearman’s rank correlations of alpha diversity measures (Shannon index, observed genera, and Pielou’s evenness index) to duration of exclusive breastfeeding (0 to 6 months) and age at introduction of complementary feeding (3 to 6 months) for compiled data from SKOT I and II. Boxes indicate 25th to 75th percentiles, with mean values marked as a line and whiskers indicating minimum and maximum values.

Danish mothers are advised to exclusively breastfeed their infants until the age of approximately 6 months and to continue partial breastfeeding until the infant is about 1 year old. It is additionally recommended to introduce complementary foods (apart from infant formula) at about the age of 6 months but not before the age of 4 months (35). As we have previously reported (25), infants in the SKOT I cohort were both exclusively and partially breastfed significantly longer than infants in the SKOT II cohort. Additionally, age at the introduction of complementary foods was significantly lower in SKOT II than in SKOT I (Table 1). Despite the fact that no infants in either of the cohorts were exclusively breastfed beyond the age of 6 months, the recorded duration of exclusive breastfeeding was associated with the relative abundance of specific bacterial taxa at the age of 9 months. This was most pronounced in SKOT I, possibly due to the longer average duration of exclusive breastfeeding in this cohort (Fig. 4A and B). However, differences in the effects of duration of exclusive breastfeeding on microbiota between cohorts were modest and not large enough to evoke detectable significant differences between the two cohorts at the age of 9 months (Fig. 1 to 3). In both cohorts, the duration of exclusive breastfeeding was negatively correlated with *Lachnospiraceae* (e.g., the genera *Dorea*, *Coprococcus*, *Blautia*, *Pseudobutyrivibrio*, and *Roseburia*) and genera within *Ruminococcaceae* (e.g., *Ruminococcus*, *Anaerotruncus*, *Oscillibacter*, *Clostridium* IV, and *Butyricicoccus*), encompassing species known to utilize plant-derived complex carbohydrates and resistant starch introduced with solid foods (36). Also, *Erysipelotrichaceae*, *Peptostreptococcaceae*, and *Eubacteriaceae* were negatively affected by the duration of exclusive breastfeeding (Fig. 4A). Positive correlations with exclusive breastfeeding were observed in both cohorts for *Bifidobacteriaceae* (*Bifidobacterium*), which are known to utilize the lactose and human milk oligosaccharides found in breast milk (37), and *Veillonellaceae* (e.g., *Veillonella* and *Megasphaera*), known lactate utilizers (38, 39). In addition, *Pasteurellaceae* (*Haemophilus*) abundances were positively correlated with the duration of exclusive breastfeeding (Fig. 4A and B). Although not significant in both cohorts, lactic acid bacteria (*Lactobacillaceae*, *Enterococcaceae*, *Streptococcaceae*) and other bacteria known to be present in human milk, like *Prevotella* (40), and on breast tissue, like *Enterobacteriaceae* (*Escherichia* and *Klebsiella*) (41), were positively correlated with duration of exclusive breastfeeding (Fig. 4A and B). At the age of 9 months, 97 infants (*n*_SKOT I_ = 59, *n*_SKOT II_ = 38) were still partially breastfed. Additionally, the estimated average daily breast milk intake at the age of 9 months was strongly correlated with gut microbiota composition and confirmed the associations obtained for the duration of exclusive breastfeeding (see Table S5 in the supplemental material). Consistently with our previous report (8), the effects of breastfeeding on microbial composition were limited at 18 months (Table S6). Some infants are fed with infant formula as a replacement or a supplement to breastfeeding for a period prior to the introduction of complementary foods. However, age at the introduction of complementary foods (range, 3 to 6 months) did not correlate with abundances of specific bacterial families at 9 months (Table S7). Furthermore, alpha diversity measures at 9 months were negatively correlated with the duration of exclusive breastfeeding, whereas age of introduction to complementary feeding was generally not correlated with alpha diversity measures, although a weak negative association with observed genera at 9 months was observed (Fig. 4C). These results suggest that breastfeeding duration, rather than the timing of introduction of complementary foods, is reflected in gut microbiota composition during late infancy, as recently proposed in a study of gut microbiome data from Swedish infants (27).

10.1128/mSphere.00069-15.7 Table S5  Spearman’s correlations of food groups to relative abundances of gut microbial families at 9 months of age when data from SKOT I and II are compiled. False-discovery-rate-corrected *P* values (*q* values) are given, and values that are <0.1 are bold. Download Table S5, DOCX file, 0.02 MB. Copyright © 2016 Laursen et al. 2016 Laursen et al. This content is distributed under the terms of the Creative Commons Attribution 4.0 International license.

10.1128/mSphere.00069-15.8 Table S6  Spearman’s rank correlations between the durations of exclusive breastfeeding and total breastfeeding and relative abundances of bacterial families at 18 months of age. Download Table S6, DOCX file, 0.02 MB. Copyright © 2016 Laursen et al. 2016 Laursen et al. This content is distributed under the terms of the Creative Commons Attribution 4.0 International license.

10.1128/mSphere.00069-15.9 Table S7  Spearman’s rank correlations between age at introduction to complementary feeding and relative abundances of bacterial families at 9 months of age. Download Table S7, DOCX file, 0.02 MB. Copyright © 2016 Laursen et al. 2016 Laursen et al. This content is distributed under the terms of the Creative Commons Attribution 4.0 International license.

### Composition of complementary diet during late infancy affects gut microbiota composition.

**FIG 5:**
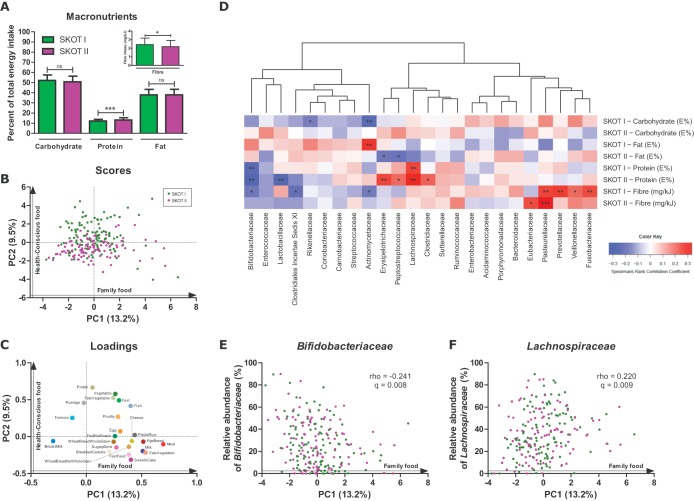
Complementary diet affects late-infancy gut microbiota composition. (A) Macronutrient intake in SKOT I and II is expressed as a mean percentage of total energy intake (E%) from carbohydrates, protein, and fat, whereas fiber intake is expressed in milligrams per kilojoule, with error bars indicating standard deviations. (B and C) PCA plot of consumption of the 23 food groups (grams per day per kilogram of body weight) for each individual in SKOT I and II (*n =* 217), resulting in principal component 1 (PC1), designated family food, and PC2, designated health-conscious food. (D) Heatmap illustrating hierarchical clustering of Spearman’s rank correlations between relative abundances of family-level gut microbes and macronutrient intake. *, *P* < 0.05; **, *P* < 0.01; ***, *P* < 0.001. (E and F) Spearman’s rank correlations between family food and relative abundances of *Bifidobacteriaceae* and *Lachnospiraceae*. *q* values are false-discovery-rate-corrected *P* values.

A validated 7-day food registration was performed by the parents when the infants were 9 months old (42). On the macronutrient level, no significant differences in fat or carbohydrate intake were observed between the two cohorts; however, protein intake was significantly higher (*P* < 0.0001, Student's *t* test) in SKOT II, while SKOT I infants had a significantly higher (*P* = 0.016, Student's *t* test) fiber intake (Fig. 5A). To capture the complete picture of the complementary diet of the infants in both cohorts at 9 months of age, we previously (25) divided the complete dietary recordings into 23 food groups (defined in Table 2). By PCA of the compiled SKOT I and II subsets of data included in this study (*n =* 217), the previously defined (25) principal components named family foods (PC1) and health-conscious food (PC2) were generated (Fig. 5B). The family foods component describes the transition from early infant foods (with low loadings of breast milk, formula, and porridge) to foods introduced during late infancy (with high loadings of meat, milk, cheese, animal fat, and rye bread). The health-conscious food component describes the amount of health-conscious food choices with low loadings of sweets/cake, sugary drinks, and fast food and high loadings of fruits, vegetables, fats (vegetable), potatoes, and fish (Fig. 5C). As previously shown (25), there was no difference between the two cohorts with respect to intake of family food (*P* = 0.481, Mann-Whitney test); however, SKOT I had significantly higher scores for health-conscious food (*P* < 0.0001, Student's *t* test with Welch's correction), corresponding to a higher intake of fruits, vegetables, and potatoes (Table 2). Clustering of Spearman’s rank correlations between macronutrient types and gut microbiota compositions revealed that bacterial groups associated with breast milk and early infant feeding, namely, *Bifidobacteriaceae*, *Enterococcaceae*, and *Lactobacillaceae*, formed a cluster characterized by negative associations with fiber as well as protein intake (Fig. 5D). The families *Erysipelotrichaceae*, *Peptostreptococcaceae*, *Lachnospiraceae*, *Clostridiaceae*, *Sutterellaceae*, and *Ruminococcaceae* formed a cluster positively associated with protein intake, while *Eubacteriaceae*, *Pasteurellaceae*, *Prevotellaceae*, *Veillonellaceae*, and *Fusobacteriaceae* were all positively associated with fiber intake (Fig. 5D). In both cohorts, fiber intake was significantly positively correlated with *Pasteurellaceae* abundance. Additionally, compiling of cohorts revealed that *Pasteurellaceae* correlated positively (*q* = 0.012) with health-conscious foods (Table S5). In both of the independent cohorts, protein intake was significantly positively correlated with *Lachnospiraceae* but significantly negatively correlated with *Bifidobacteriaceae*, probably reflecting the amount of complementary food in the infant’s diet (Fig. 5D). Indeed, the family food dietary pattern, reflecting progression of an infant’s diet toward family foods, was negatively associated with *Bifidobacteriaceae* abundance (Fig. 5E) but positively associated with *Lachnospiraceae* abundance (Fig. 5F). Further, family food correlated negatively (*q* = 0.019) with *Enterococcaceae* abundance and positively correlated (*q* = 0.009) with *Sutterellaceae* abundance (Table S5). These results suggest that the progression from early infant food to family foods with higher protein and fiber contents is the major driver of gut microbial changes during late infancy.

**TABLE 2:** Food group definitions, average intake, and correlation to gut microbial alpha diversity at 9 months of age in SKOT I and SKOT II^a^

Food group	Definition	Avg intake (g/day/kg body wt)			Spearman correlation to Shannon diversity (SKOT I + SKOT II)		
		SKOT I (*n* = 114)	SKOT II (*n* = 103)	SKOT I + SKOT II (*n* = 217)	rho	*P* value	*q* value
Porridge	Cereal gruel and porridge (homemade or already prepared)	18.21	15.03	16.70	0.143	**0.039**	0.149
Breakfast cereals	Oatmeal, muesli, Cornflakes, sugar puffs, and sugary cereals	0.13	0.17	0.15	−0.044	0.525	0.636
Wheat bread, whole grain	Grainy bread and crisp bread	1.05	1.73	1.38	−0.032	0.644	0.705
Wheat bread, no whole grain	White bread and biscuits	0.57	0.89	0.72	0.072	0.299	0.416
Rye bread	Rye bread with and without seeds	1.28	1.40	1.34	0.235	**<0.001**	**0.004**
Pasta/rice	Pasta and rice	0.90	0.97	0.93	0.120	0.083	0.192
Potato	Potatoes that were boiled, baked, mashed, or prepared in potato salad	4.48	1.51	3.07	0.073	0.294	0.416
Fruit	Fresh fruit, fresh berries, and fruit porridge, soup, or compote (homemade or already prepared)	12.47	8.23	10.46	0.111	0.110	0.231
Vegetable	All vegetables eaten raw, cooked, or mashed alone or in a dish	6.84	3.26	5.14	0.134	0.053	0.153
Fish	All fish and fish products eaten as a sandwich spread or in a dish	0.81	0.71	0.76	0.122	0.080	0.192
Meat	All meat and meat products eaten as a sandwich spread or in a dish, except poultry and fish	1.63	1.74	1.68	0.274	**<0.0001**	**<0.001**
Poultry	All poultry and poultry products eaten as a sandwich spread or in a dish	0.43	0.35	0.39	0.039	0.578	0.664
Egg	All egg and egg products eaten as a sandwich spread or in a dish	0.16	0.14	0.15	0.003	0.968	0.968
Fats (animal)	Butter, spreadable butter, and sauce made from butter	0.98	1.01	1.00	0.078	0.264	0.416
Fats (vegetable)	Oil, margarine, mayonnaise, remoulade, ketchup, and low-fat sauce	0.56	0.38	0.47	0.138	**0.046**	0.153
Cheese	All cheese and cheese products eaten as a sandwich spread or in a dish	0.55	0.65	0.59	0.296	**<0.0001**	**<0.0001**
Milk	All milk and milk products eaten alone or in a dish, except human milk or infant formula	12.95	17.90	15.30	0.156	**0.024**	0.111
Formula	Infant formula and follow-up formula	32.71	27.77	30.37	0.071	0.308	0.416
Breast milk	Human milk from the mother	11.62	8.06	9.93	−0.366	**<0.0001**	**<0.0001**
Fruit/nut/snack	Cereal bar, nuts, almonds, dried fruit, fruit spread, jam, honey, peanut butter, and seeds	0.31	0.16	0.24	0.051	0.468	0.597
Sweets/cake	Ice cream, chocolate, licorice, soufflé, croissant, Danish pastry, cookies, cream cake, pancake, and cream puff (mix of light/not light versions)	0.21	0.16	0.19	0.086	0.218	0.412
Sugary drink	Soda, juice, lemonade, chocolate milk, milk shake, and yogurt drink (mix of light/not light versions)	1.12	0.39	0.78	−0.015	0.826	0.863
Fast food	Fried potatoes, French fries, hot dogs, pizza, burgers, spring rolls, and chips	0.59	0.50	0.55	0.083	0.233	0.412

a The food group definitions are from reference 25. Gut microbial alpha diversity is the Shannon index. Significant *P* and *q* values are highlighted in bold. Data from 10 individuals from SKOT II were missing.

### Progression toward family foods increases the alpha diversity of the infant gut microbiota.

**FIG 6:**
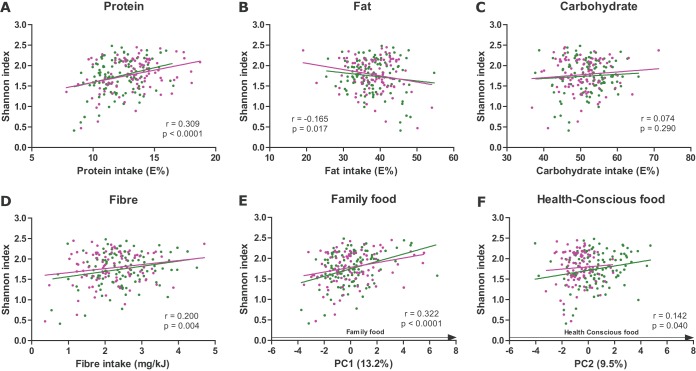
The transition to family foods with higher protein and fiber content correlates with increased gut microbiota diversity. Pearson correlations of gut microbial alpha diversity (Shannon index) with macronutrient intake (A to D) and the dietary patterns family food and health-conscious food (E and F) at 9 months of age (SKOT I, green; SKOT II, purple).

Next, we investigated how the complementary diet affected the gut microbial alpha diversity. At the age of 9 months, protein and fiber intake was significantly positively associated with the Shannon index, while fat intake was negatively correlated with the Shannon index. No significant association was observed between carbohydrate intake and the Shannon index (Fig. 6A to D). Both the dietary pattern family food and to a lesser extent the dietary pattern health-conscious food were significantly positively associated with the Shannon index (Fig. 6E and F). Similar associations were observed between nutrient groups and the other alpha diversity measures, namely, observed genera and Pielou’s evenness index (Fig. S1 to 2), indicating that both microbial richness and evenness are affected by the complementary diet. These correlations reflect that foods with high loadings in the family food dietary pattern (Fig. 5C), including cheese, meat, and rye bread, were positively associated with alpha diversity, while breast milk consumption at the age of 9 months was negatively correlated with alpha diversity (Table 2). After correction for multiple testing, the remaining 19 food groups did not correlate significantly with alpha diversity (Table 2), indicating that the progression of an infant’s diet toward family foods, characterized by the transition from breastfeeding to more nutritionally diverse family foods rich in fiber and proteins, is the main driver of gut microbial alpha diversity development.

10.1128/mSphere.00069-15.1 Figure S1  The transition to family foods with higher protein and fiber content correlates with gut microbial richness. Pearson correlations of gut microbial richness (observed genera) with macronutrient intake (A to D) and the dietary patterns family food and health-conscious food (E and F) at 9 months of age (SKOT I, green; SKOT II, purple). Download Figure S1, EPS file, 2.6 MB. Copyright © 2016 Laursen et al. 2016 Laursen et al. This content is distributed under the terms of the Creative Commons Attribution 4.0 International license.

## DISCUSSION

Elucidating the influence of dietary factors on the development of the gut microbiota during early life is necessary in order to understand potential links between childhood diet and microbiota-associated disease risks later in life. Indeed, the early life period is considered a window of opportunity for microbial manipulation, due to the relatively unstable configuration of the incompletely developed gut microbiota (9). This study reveals that maternal obesity *per se* does not affect the development of the gut microbiota during the complementary feeding period. Rather, the gradual process leading to the establishment of a complex microbiota, which occurs during infancy and early childhood, is connected primarily to the transition from breastfeeding to family-like foods rich in fiber and protein and apparently independent of other potential effects, such as lifestyle and genetic disposition related to maternal obesity. Until now, only a few studies have investigated potential links between maternal obesity and offspring microbiota (43, 44). Collado et al showed that levels of *Staphylococcus* and *Bacteroides* were significantly higher in infants (aged 1 and 6 months) of overweight mothers (BMI > 25) than in infants of normal-weight mothers, whereas levels of *Bifidobacterium* spp. were lower (43). Galley et al. found higher alpha diversity and lower beta diversity in children (aged 18 to 27 months) of obese mothers than in children of nonobese mothers but primarily in a subgroup of individuals of higher socio-economic status. These findings were not confirmed by the present study. Previously reported evidence has revealed differences in gut microbiotas between lean and obese individuals (16, 45); however, the infants/children in the present study were not obese at the sampling points. Nonetheless, it is plausible that the relatively late sampling point (9 months) used in this study explains the lack of observed differences in the infants, since potential initial differences in maternally transferred gut microbes may be overruled by the effect of breastfeeding and complementary diet by that age. Regardless of this, we find it remarkable that the communities developed so similarly in the two different cohorts (discordant for maternal obesity), which were independently sampled and originated from infants of different socio-economic backgrounds (Table 1). Despite differences between cohorts in breastfeeding duration and complementary feeding practices (Table 1), these differences did not cause significant differences in gut microbiotas between cohorts within the given sensitivity and taxonomic resolution of our method. Importantly though, the high similarity between cohorts enabled us to cross-validate associations. We observed that *Lachnospiraceae*, *Ruminococcaceae*, *Eubacteriaceae*, *Rikenellaceae*, and *Sutterellaceae* constitute the major bacterial families that increase in abundance from infancy to the toddler age (early childhood) and vice versa for *Bifidobacteriaceae*, *Actinomycetaceae*, *Veillonellaceae*, *Enterobacteriaceae*, *Lactobacillaceae*, *Enterococcaceae*, *Clostridiales incertae* *sedis* XI, *Carnobacteriaceae*, and *Fusobacteriaceae*, which is largely in agreement with previous studies (27, 29, 30). In contrast to what was anticipated, we found limited effect of C section and gestational age on gut microbiota, but this is probably explained by the fact that our first point of sampling was not until the age of 9 months, at which time such effects are likely to have been diluted by other influencing factors, including breastfeeding and complementary feeding. The relative abundance of gut bacterial taxa as well as alpha diversity measures at the age of 9 months was explained by breastfeeding duration rather than by time of introduction to complementary (solid) foods, in line with what was recently suggested by Bäckhed et al. (27). Specifically, we found *Bifidobacterium*, *Veillonella*, *Megasphaera*, *Haemophilus*, and members of lactic acid bacteria and the *Enterobacteriaceae* to be positively affected by breastfeeding duration, while the reverse was true for members of *Lachnospiraceae* and *Ruminococcaceae*, known to utilize complex carbohydrates (36). Further, at the age of 9 months, the level of progression toward family foods, reflected in a higher consumption of meat, cheese, and rye bread, rich in fiber and protein, was clearly associated with gut microbiota composition and alpha diversity measures. Particularly, *Lachnospiraceae* abundance increased with the amount of family foods in the diet, while the opposite was found for *Bifidobacteriaceae*. This probably reflects the shift from breastfeeding associated with *Bifidobacteriaceae* to late-infancy foods, resembling the food of the family with higher fiber and protein content, which selects for species within the *Lachnospiraceae*. The fact that Shannon diversity, richness, and evenness correlate with the transition to family foods indicates both that the number of different microbes increases and that their mutual distribution evens with the progression toward family foods. This might be interpreted as a sign of increased gut microbial stability. In adults, whey protein and meat were recently found to be positively correlated with gut microbial alpha diversity (46), and a high fiber content (e.g., arabinoxylans) of rye bread has been shown to increase butyrate concentration in feces (47) and plasma (48) and may also contribute to an increased alpha diversity of the microbiota by overall increasing the diverse group of bacterial butyrate producers (49). Consumption of both protein and fiber increases during the complementary feeding period and might therefore represent new energy sources for gut microbes at an infant age of 9 months. This may result in selective advantages for specific microbes to establish in the gut, which will increase alpha diversity.

Although we also investigated associations between anthropometrical data and gut microbiota features, we found no significant correlations after correction for multiple testing. This might be explained by the relative healthy conditions of infants included in the SKOT cohorts. However, the body of recent evidence for a relationship between early gut microbial diversity and metabolic, autoimmune, and allergic diseases (9) emphasizes that our findings are relevant for elucidation of the relationship between complementary diet, gut microbiota establishment, and disease risk. In summary, we conclude that maternal obesity *per se* does not impact gut microbial changes during late infancy and early childhood substantially. Rather, variation in late-infancy gut microbiota is affected by breastfeeding, complementary diet, and the transition toward family foods with high protein and fiber contents, such as meat, cheese, and rye bread. An increased understanding of the influence of the complementary diet on the development and establishment of the infant gut microbiota provides us with tools to tailor a beneficial progression of our intestinal microbial consortium.

## MATERIALS AND METHODS

### SKOT cohorts.

The present study used samples from the SKOT cohorts, in which 311 (SKOT I) and 184 (SKOT II) Danish children were followed for the first 3 years after birth, with the overall aim of investigating relationships between early diet, growth development, and later disease risks, especially obesity and metabolic syndrome. The study protocols were approved by the Committees on Biomedical Research Ethics for the Capital Region of Denmark (H-KF-2007-0003 and H-3-2010-122). In SKOT I, infants from a random sample of mothers were recruited, whereas in SKOT II, only infants of obese mothers (BMI > 30 kg/m^2^) were recruited (25). Inclusion criteria in both cohorts were single birth and full-term delivery, an absence of chronic illness, and an age of 9 months ± 2 weeks at the first visit. Recruitment of participants for SKOT I was done by postal invitations sent to 2,211 randomly selected mothers of infants based on the National Civil Registration System (24). A positive response was obtained from 330 (15%), but 19 dropped out before the first examination; thus, 311 individuals were included in the cohort. With exclusions of individuals with fecal samples taken at only one of the two time points (9 or 18 months of age), individuals using antibiotics, or individuals for which we had inappropriately stored fecal samples (e.g., samples stored at room temperature, for which the storage time was >24 h, or that defrosted before delivery to the university), a randomly selected subset of 114 samples was used in the present study. In SKOT II, 184 infants of the invited 208 obese pregnant women participating in the TOP (*t*reatment of *o*bese *p*regnant women) intervention study were recruited (50). Using the same exclusion criteria for fecal samples that were used in SKOT I, a subset of 113 infants was included in this study. Collection of data was carried out from 2007 to 2010 and from 2011 to 2014 for SKOT I and SKOT II, respectively. Official Danish guidelines for infant feeding did not change during this period. Participants in both cohorts were examined at 9 months (±2 weeks), 18 months (±4 weeks), and 36 months (±12 weeks) of age, and fecal samples and information on body composition, food questionnaires, and background interviews were collected at these examinations during the study. Several studies, not including microbiota assessments, have been published previously on these cohorts (24, 25). For SKOT I, one paper reporting on the microbiota assessed by a qPCR-based approach (8) and one study reporting on the relation between microbiota data from SKOT I and nondietary factors affecting gut microbiota and prevalence of atopic symptoms (51) are available.

### Anthropometry and body composition.

Birth weight and length measurements were taken by midwives and obtained from health records. Weight, length, waist circumference, and subscapularis skinfold thickness measurements at 9 and 18 months were taken at the Department of Nutrition, Exercise and Sports, University of Copenhagen, by trained research staff. Using a digital scale (at 9 months, SartoriusIP 65 [Sartorius AG, Göttingen, Germany]; at 18 months, Lindeltronic 8000 [Samhall Lavi AB, Kristianstad, Sweden]), weight was measured, without clothes, to the nearest 0.1 kg. Recumbent length was a mean of three measurements carried out with a digital measuring board (Force Technology, Brøndby, Denmark), which made readings to the nearest 0.01 cm. Skinfold thickness was measured to the nearest 0.1 mm by a Harpenden skinfold caliper (Chasmors Ltd., London, United Kingdom), and we used the mean of three measurements. Recumbent waist circumference was measured to the nearest millimeter at the level of the umbilicus with a nonstretchable tape measure (Lasso; Child Growth Foundation, London, United Kingdom). Weight, length, subscapularis skinfold thickness, and BMI were converted to *Z* scores, with the World Health Organization growth standards used as a reference and with the software program World Health Organization Anthro (52).

### Food questionnaire.

As described previously (25), the infant diet was recorded by parents at the age of 9 months using validated 7-day food records (42). Portion sizes were estimated with household measures and food photograph series and noted in a precoded food diary. All intakes of energy, nutrients, and food items recorded in the precoded food record were calculated for each individual using the software system GIES (version 1.000d; National Food Institute, Søborg, Denmark), a system developed at the National Food Institute, Technical University of Denmark, and the Danish Food Composition Databank (version 7; National Food Institute [http://www.Foodcomp.dk]). Quality control was carried out by trained research staff before data were entered in the database. Possible over- and underreporters were identified on the basis of the estimated daily energy requirement of 338 kJ/kg for both genders, an average between the 6- and 12-month estimates and cutoff values of ±46% (53). The food groups (Table 2) were selected on the basis of nutritional knowledge in an attempt to cover most aspects of the official recommendations, nutrition evidence, and typical infant diet in Denmark. Food groups were named with a short, compressed description, such as “RyeBread” and “SugaryDrink.” Intake of breast milk was calculated as the number of breastfeedings per day, using a rough estimate of 99 g per feeding (54). The intake (grams/day) of all food groups was divided by total body weight (in kilograms) for each participant.

### Information extracted from parental background interviews.

Information on sex, socio-economics (work situation, education level, and household income), prevalence of C section, gestational age at birth, prior use of antibiotics and other medication, durations of exclusive and total breastfeeding, and age of introduction to complementary feeding (Table 1 and see Tables S2 to S4, S6, and S7 in the supplemental material) were collected from background interviews with parents at the 9- and 18-month visits. Use of antibiotics was recorded 14 days prior to the 9- and 18-month visits. Exclusive breastfeeding was defined as receiving only breast milk, water, and vitamins.

### Fecal samples, DNA extraction, and PCR amplification of the V3 region of the 16S rRNA gene.

Fecal samples obtained at 9 and 18 months of age were freshly delivered on the morning of visitation or had been stored in the participant’s home, in provided freezer containers, either in the freezer (−18°C) or in the refrigerator (4°C) for maximally 24 h before delivery to the University of Copenhagen’s Department of Nutrition, Exercise and Sports, where they were stored at −80°C until DNA extraction. Samples were randomized across cohorts (*n*_SKOT I_ = 10, *n*_SKOT II_ = 10) for each DNA extraction round (*n*_total_ = 20). DNA was extracted (12855-100 PowerLyzer PowerSoil DNA isolation kit; Mo Bio) from 250 mg feces according to the protocol provided by the manufacturer with minor modifications: bead beating was performed at 30 cycles/s for 10 min (Retsch MM 300 mixer mill), and the initial centrifugation steps were performed at 10,000 × *g* for 3 min, as recommended for clay matter. DNA quantity and quality were measured by the Qubit double-stranded-DNA (dsDNA) BR assay (Invitrogen; Q32850) and with a NanoDrop 1000 (Thermo Scientific), respectively, yielding on average 32.7 ± 21.4 ng/µl DNA with an *A*_260_/*A*_280_ equal to 1.81 ± 0.12 and an *A*_260_/*A*_230_ equal to 1.60 ± 0.39. The PCR amplification of the V3 region of the 16S rRNA gene was performed with 5 ng community DNA as the template, using 0.2 µl Phusion high-fidelity (HF) DNA polymerase (Fisher Scientific; F-553L), 4 µl HF buffer, 0.4 µl deoxynucleoside triphosphate (dNTP) (10 mM of each base), 1 µM forward primer (PBU [primer bacterial universal] 5′-A-adapter-TCAG-barcode-CCTACGGGAGGCAGCAG-3′) and 1 µM reverse primer (PBR [primer bacterial reverse] 5′-trp1-adapter-ATTACCGCGGCTGCTGG-3′) in a 20-µl total reaction volume. Both primers include sequencing adaptors, and the forward primer additionally includes a unique 10- to 12-bp barcode (IonXpress barcode adapters). The PCR program included 30 s at 98°C, 24 cycles of 15 s at 98°C and 30 s at 72°C, and then 5 min at 72°C. The PCR product was purified by use of HighPrep PCR magnetic beads (MagBio; AC-60005) with the 96-well magnet stand (MagBio; MyMag 96), according to the prescribed procedure. DNA quantity was measured using the Qubit dsDNA HS assay (Invitrogen; Q32851), and samples were pooled to obtain equimolar libraries containing up to 90 samples (randomized across cohorts and age) in each library.

### DNA sequencing and data handling.

Sequencing of the 16S rRNA gene libraries was performed using the Ion OneTouch and Ion personal genome machine (PGM) systems with an Ion 318 chip kit, generating 5 to 7 million reads per chip with a median length of 180 bp. Sequencing data were imported into CLC Genomic Workbench (version 7.0.3, CLC bio; Qiagen, Aarhus, Denmark), reads were quality controlled, demultiplexed, and trimmed to remove low-quality sequences (*P*_base-calling error_ = 0.05), ambiguous nucleotides (maximum of 2 allowed), primers, and barcodes and to discard reads below 110 bp and above 180 bp. The sorted and trimmed FASTA files were run through the Ribosomal Database Project classifier (55), with a bootstrap cutoff of 50% as recommended for sequences shorter than 250 bp (56). Chimera removal was not performed, since short amplicon length and a low number of PCR cycles reduces chimera prevalence (57). The total number of reads for each sample was on average 47,544 ± 18,656 (range, 12,749 to 121,070) and was used to calculate the relative abundances of bacterial taxons at the phylum (99.8% classified), family (98.3% classified), and genus (75.2% classified) levels. In the further analysis, a cutoff of 0.01% in mean relative abundance at either 9 or 18 months was set. Based on the detection limit (1 read), a threshold was set to 0.001% (~0.48 reads) and samples with zero reads for a given bacterial taxon were assigned this value. Based on reads assigned to the genus level (average, 36,012 ± 16,221; range, 4,384 to 92,980), 8 samples were excluded due to low depth (<10,000 reads) for calculation of alpha and beta diversity measures. In the remaining 446 samples, sequences were rarefied (average of 100 subsamplings) to 10,000 reads/sample. Binary Bray-Curtis dissimilarity and alpha diversity measures (Shannon index, number of observed genera, and Pielou’s evenness index) were calculated for each individual in each cohort at the ages of 9 and 18 months using the R package vegan. Based on principal-coordinate analysis (PCoA) of Bray-Curtis dissimilarities, the distances to group centroids were used as measures of beta diversity, using the function betadisper within vegan.

### Statistical tests and correlations.

Correlation analyses and statistical tests were done with the GraphPad Prism software (version 5.0.3; GraphPad Software Inc., La Jolla, CA) and R (version 3.1.0, R Core Team 2014 R:A language and environment for statistical computing; R Foundation for Statistical Computing, Vienna, Austria). Principal-component/coordinate analyses (prcomp/betadisper) and heatmaps (heatmap.2) were performed in R using the packages ggbiplot, gplot, and vegan. Normal distribution of data was evaluated by the Shapiro-Wilk normality test and visual inspection of histograms. Cohort characteristics were compared by Fisher’s exact test/chi-square test for categorical data and an unpaired *t* test or the Mann-Whitney test for continuous data. A paired Wilcoxon signed-rank test or paired *t* test was used to examine the changes in alpha diversity measures and bacterial composition across time, whereas the Mann-Whitney test or unpaired *t* test was used to compare measures of alpha diversity and bacterial composition between cohorts at the ages of 9 and 18 months. Turkey’s honestly significant differences test was used to evaluate differences in beta diversity across time and cohorts.

Spearman’s rank test/Pearson’s correlations and the unpaired *t* test/Mann-Whitney test were used to investigate associations between gut bacterial composition and alpha diversity measures with birth mode, gestational age at birth, prior use of antibiotics, duration of exclusive/total breastfeeding, age at introduction to complementary feeding, complementary diet at 9 months, and anthropometrics at 9 and 18 months. When indicated, false discovery rate-corrected (58) *P* values (*q* values) were applied to correct for multiple testing, with a threshold of 0.05.

### Accession numbers.

Sequencing data are deposited in NCBI’s Sequence Read Archive with the accession number SRP052851 under BioProject number PRJNA273694.

10.1128/mSphere.00069-15.2 Figure S2  The transition to family foods with higher protein and fiber content correlates with gut microbial evenness. Pearson correlations of gut microbial evenness (Pielou’s evenness index) with macronutrient intake (A to D) and the dietary patterns family food and health-conscious food (E and F) at 9 months of age (SKOT I, green; SKOT II, purple). Download Figure S2, EPS file, 2.6 MB. Copyright © 2016 Laursen et al. 2016 Laursen et al. This content is distributed under the terms of the Creative Commons Attribution 4.0 International license.
