# Supplementary material for: Infant Gut Microbiota Development Is Driven by Transition to Family Foods Independent of Maternal Obesity
Source: mSphere. 2016 Feb 10;1(1):e00069-15. doi: 10.1128/mSphere.00069-15 (PMC4863607; doi:10.1128/mSphere.00069-15)
Supplement: Table S1 [file sph001162013st3.docx]

| ***Family***  *Genus* | | **SKOT I** | | | **SKOT II** | | |
| --- | --- | --- | --- | --- | --- | --- | --- |
|  |  | **Mean relative abundance (%)** | | ***q*-value^a^** | **Mean relative abundance (%)** | | ***q*-value^a^** |
|  |  | **9 months** | **18 months** |  | **9 months** | **18 months** |  |
| ***Lachnospiraceae*** | | **34.82** | **47.22** | **9.05E-06** | **34.87** | **45.15** | **3.84E-05** |
|  | *Blautia* | 8.90 | 13.52 | 8.03E-04 | 8.81 | 12.59 | 4.04E-03 |
|  | *Roseburia* | 2.38 | 5.43 | 4.51E-06 | 3.46 | 4.78 | 1.28E-02 |
|  | *Pseudobutyrivibrio* | 1.22 | 3.55 | 9.34E-07 | 0.73 | 3.36 | 2.42E-08 |
|  | *Dorea* | 0.52 | 1.34 | 6.13E-06 | 0.32 | 0.95 | 3.91E-08 |
|  | *Clostridium XlVa* | 0.43 | 0.08 | 8.91E-07 | 0.24 | 0.13 | ns |
|  | *Anaerostipes* | 0.42 | 0.14 | ns | 0.31 | 0.12 | 2.93E-03 |
|  | *Coprococcus* | 0.07 | 0.32 | 3.34E-11 | 0.04 | 0.33 | 5.46E-11 |
|  | *Moryella* | 0.02 | 0.04 | 8.15E-06 | 0.02 | 0.04 | 3.37E-05 |
|  | *Clostridium XlVb* | 0.01 | 0.02 | 1.04E-02 | <0.01 | 0.01 | 2.22E-03 |
|  | *Unclassified Lachnospiraceae* | 19.36 | 21.68 | - | 19.77 | 21.85 | - |
| ***Ruminococcaceae*** | | **6.43** | **19.15** | **8.15E-12** | **5.81** | **18.19** | **3.89E-12** |
|  | *Faecalibacterium* | 4.94 | 14.85 | 6.92E-11 | 4.16 | 13.91 | 1.57E-10 |
|  | *Gemmiger* | 0.57 | 1.87 | 8.91E-07 | 0.48 | 1.92 | 3.91E-08 |
|  | *Ruminococcus* | 0.43 | 0.53 | 3.11E-04 | 0.60 | 0.63 | 8.18E-03 |
|  | *Butyricicoccus* | 0.08 | 0.42 | 4.64E-11 | 0.12 | 0.34 | 3.06E-09 |
|  | *Anaerotruncus* | 0.02 | 0.01 | 8.03E-04 | 0.02 | <0.01 | ns |
|  | *Oscillibacter* | 0.01 | 0.07 | 7.14E-08 | <0.01 | 0.05 | 1.31E-09 |
| ***Eubacteriaceae*** | | **0.04** | **0.20** | **5.93E-07** | **0.04** | **0.17** | **1.12E-06** |
|  | *Eubacterium* | 0.04 | 0.19 | 8.91E-07 | 0.03 | 0.16 | 3.48E-06 |
| ***Clostridiaceae*** | | **1.07** | **1.00** | **ns** | **1.54** | **1.40** | **ns** |
|  | *Clostridium sensu stricto* | 0.24 | 0.05 | 1.66E-08 | 0.37 | 0.11 | 1.29E-10 |
|  | *Sarcina* | 0.08 | 0.18 | ns | 0.08 | 0.18 | 7.39E-03 |
| ***Veillonellaceae*** | | **5.85** | **2.88** | **9.02E-03** | **5.05** | **2.42** | **5.24E-04** |
|  | *Veillonella* | 4.81 | 0.44 | 2.23E-11 | 4.21 | 0.52 | 2.31E-12 |
|  | *Megasphaera* | 0.39 | 0.16 | 9.00E-08 | 0.31 | 0.07 | 6.71E-09 |
|  | *Dialister* | 0.22 | 1.94 | 2.23E-11 | 0.17 | 1.63 | 4.85E-12 |
| ***Enterococcaceae*** | | **0.81** | **0.18** | **5.05E-11** | **0.73** | **0.07** | **3.73E-11** |
|  | *Enterococcus* | 0.81 | 0.18 | 4.64E-11 | 0.72 | 0.07 | 5.46E-11 |
| ***Lactobacillaceae*** | | **0.45** | **0.09** | **4.93E-03** | **0.95** | **0.25** | **1.84E-03** |
|  | *Lactobacillus* | 0.42 | 0.09 | 4.54E-03 | 0.92 | 0.25 | 4.04E-03 |
|  | *Pediococcus* | 0.03 | <0.01 | ns | 0.02 | <0.01 | 7.39E-03 |
| ***Erysipelotrichaceae*** | | **3.28** | **1.52** | **2.71E-03** | **1.81** | **1.24** | **ns** |
|  | *Clostridium XVIII* | 1.86 | 0.57 | 1.00E-04 | 1.17 | 0.68 | 4.92E-02 |
|  | *Erysipelotrichaceae incertae sedis* | 1.11 | 0.19 | 4.64E-11 | 0.48 | 0.19 | 1.35E-07 |
| ***Clostridiales Incertae Sedis XI*** | | **0.09** | **<0.01** | **4.92E-02** | **0.03** | **<0.01** | **4.76E-03** |
|  | *Finegoldia* | 0.08 | <0.01 | 5.67E-04 | 0.01 | <0.01 | 1.03E-02 |
| ***Carnobacteriaceae*** | | **0.02** | **<0.01** | **1.88E-06** | **0.02** | **0.01** | **3.84E-05** |
|  | *Granulicatella* | 0.02 | <0.01 | 8.70E-05 | 0.02 | 0.01 | 5.78E-04 |
| ***Bifidobacteriaceae*** | | **25.55** | **10.61** | **1.11E-08** | **29.52** | **12.66** | **2.41E-10** |
|  | *Bifidobacterium* | 24.91 | 10.26 | 1.07E-08 | 28.93 | 12.25 | 2.91E-10 |
| ***Coriobacteriaceae*** | | **2.88** | **3.55** | **1.63E-02** | **3.67** | **4.18** | **ns** |
|  | *Collinsella* | 2.43 | 3.17 | 9.66E-03 | 3.24 | 3.79 | ns |
| ***Actinomycetaceae*** | | **0.05** | **0.02** | **1.31E-08** | **0.08** | **0.03** | **8.17E-11** |
|  | *Actinomyces* | 0.05 | 0.02 | 1.66E-08 | 0.08 | 0.03 | 1.29E-10 |
| ***Prevotellaceae*** | | **1.21** | **1.09** | **ns** | **0.67** | **1.39** | **1.18E-02** |
|  | *Paraprevotella* | <0.01 | 0.02 | 3.10E-02 | <0.01 | 0.03 | 2.35E-03 |
| ***Porphyromonadaceae*** | | **0.58** | **0.38** | **ns** | **0.54** | **0.80** | **1.78E-03** |
|  | *Parabacteroides* | 0.53 | 0.22 | ns | 0.38 | 0.60 | 2.01E-02 |
|  | *Barnesiella* | <0.01 | 0.10 | 4.07E-04 | 0.04 | 0.14 | 2.63E-03 |
|  | *Odoribacter* | <0.01 | 0.03 | 2.96E-05 | 0.02 | 0.04 | 1.39E-03 |
| ***Rikenellaceae*** | | **0.24** | **0.66** | **1.80E-07** | **0.11** | **0.87** | **8.17E-11** |
|  | *Alistipes* | 0.24 | 0.66 | 2.06E-07 | 0.11 | 0.87 | 1.34E-10 |
| ***Enterobacteriaceae*** | | **5.15** | **1.21** | **7.16E-11** | **3.73** | **0.81** | **8.17E-11** |
|  | *Escherichia/Shigella* | 3.73 | 1.12 | 4.93E-10 | 2.56 | 0.74 | 2.91E-10 |
|  | *Klebsiella* | 1.05 | <0.01 | 7.40E-13 | 0.80 | 0.02 | 1.34E-10 |
|  | *Salmonella* | 0.04 | 0.02 | 4.28E-02 | 0.00 | <0.01 | 5.70E-03 |
|  | *Cronobacter* | 0.03 | <0.01 | 2.38E-04 | 0.01 | <0.01 | 8.64E-06 |
|  | *Raoultella* | 0.02 | <0.01 | 3.46E-03 | 0.02 | 0.01 | 3.96E-04 |
|  | *Kluyvera* | 0.02 | <0.01 | 3.11E-04 | 0.02 | <0.01 | 9.90E-06 |
|  | *Shimwellia* | 0.02 | <0.01 | 9.67E-05 | 0.02 | <0.01 | 1.11E-06 |
| ***Pasteurellaceae*** | | **0.16** | **0.26** | **ns** | **0.14** | **0.17** | **1.16E-02** |
|  | *Haemophilus* | 0.15 | 0.24 | ns | 0.12 | 0.16 | 8.15E-03 |
| ***Sutterellaceae*** | | **0.11** | **0.15** | **3.95E-03** | **0.06** | **0.21** | **3.22E-07** |
|  | *Sutterella* | 0.07 | 0.12 | 6.42E-03 | 0.05 | 0.14 | 5.78E-04 |
|  | *Parasutterella* | 0.04 | 0.03 | ns | 0.02 | 0.07 | 8.98E-05 |
| ***Fusobacteriaceae*** | | **0.02** | **0.01** | **8.24E-04** | **0.03** | **0.01** | **2.39E-02** |
|  | *Fusobacterium* | 0.02 | <0.01 | 3.71E-04 | 0.03 | 0.01 | 8.15E-03 |

a) q-values indicate the False Discovery Rate corrected p-values of paired Wilcoxon rank sum tests of relative abundances at 9 months versus 18 months.
