# Supplementary material for: Infant Gut Microbiota Development Is Driven by Transition to Family Foods Independent of Maternal Obesity
Source: mSphere. 2016 Feb 10;1(1):e00069-15. doi: 10.1128/mSphere.00069-15 (PMC4863607; doi:10.1128/mSphere.00069-15)
Supplement: Table S2 [file sph001162013st4.docx]

|  | **SKOT I** | | | |  | **SKOT II** | | | |
| --- | --- | --- | --- | --- | --- | --- | --- | --- | --- |
|  | **C-section (n=15)** | **Vaginal delivery (n=98)** | **p-value^a^** | **q-value^b^** |  | **C-section (n=35)** | **Vaginal delivery (n=69)** | **p-value^a^** | **q-value^b^** |
| **Alpha diversity** |  |  |  |  |  |  |  |  |  |
| Shannon index (mean ± sd) | 1.73 ± 0.54 | 1.75 ± 0.39 | 0.850 | - |  | 1.78 ± 0.40 | 1.77 ± 0.38 | 0.903 | - |
| Observed genera (mean ± sd) | 33.60 ± 7.14 | 34.38 ± 6.78 | 0.526 | - |  | 34.87 ± 7.53 | 35.10 ± 6.62 | 0.877 | - |
| Pielou’s evenness index (mean ± sd) | 0.50 ± 0.15 | 0.50 ± 0.10 | 0.957 | - |  | 0.51 ± 0.10 | 0.50 ± 0.10 | 0.823 | - |
| **Family level**  **Mean relative abundance (%)** |  | |  |  |  |  | |  |  |
| *Lachnospiraceae* | 41.492 | 34.056 | 0.212 | 0.632 |  | 35.772 | 36.041 | 0.889 | 0.889 |
| *Bifidobacteriaceae* | 22.423 | 26.068 | 0.252 | 0.632 |  | 30.734 | 27.901 | 0.379 | 0.675 |
| *Bacteroidaceae* | 5.033 | 6.630 | 0.357 | 0.632 |  | 3.073 | 7.124 | **0.003** | **0.072** |
| *Ruminococcaceae* | 8.496 | 6.185 | 0.749 | 0.893 |  | 5.126 | 6.576 | 0.430 | 0.688 |
| *Veillonellaceae* | 2.719 | 6.348 | 0.182 | 0.632 |  | 6.433 | 4.255 | 0.075 | 0.300 |
| *Enterobacteriaceae* | 3.818 | 5.347 | 0.350 | 0.632 |  | 3.145 | 2.924 | **0.036** | 0.246 |
| *Coriobacteriaceae* | 3.557 | 2.797 | 0.980 | 0.980 |  | 2.910 | 4.309 | **0.041** | 0.246 |
| *Erysipelotrichaceae* | 2.829 | 3.333 | 0.306 | 0.632 |  | 2.421 | 1.574 | 0.250 | 0.600 |
| *Streptococcaceae* | 1.547 | 1.315 | 0.724 | 0.893 |  | 2.088 | 1.247 | 0.889 | 0.889 |
| *Peptostreptococcaceae* | 1.758 | 1.312 | 0.395 | 0.632 |  | 1.506 | 1.454 | 0.640 | 0.827 |
| *Clostridiaceae* | 1.309 | 1.045 | 0.781 | 0.893 |  | 2.162 | 1.319 | 0.237 | 0.600 |
| *Prevotellaceae* | 0.182 | 1.384 | 0.320 | 0.632 |  | 0.092 | 1.046 | 0.859 | 0.889 |
| *Enterococcaceae* | 1.857 | 0.625 | 0.186 | 0.632 |  | 0.562 | 0.833 | 0.520 | 0.757 |
| *Lactobacillaceae* | 0.089 | 0.512 | 0.087 | 0.632 |  | 1.364 | 0.711 | 0.699 | 0.839 |
| *Porphyromonadaceae* | 0.175 | 0.650 | 0.134 | 0.632 |  | 0.443 | 0.629 | **0.017** | 0.204 |
| *Rikenellaceae* | 0.080 | 0.266 | 0.826 | 0.901 |  | 0.166 | 0.093 | 0.852 | 0.889 |
| *Pasteurellaceae* | 0.088 | 0.175 | 0.703 | 0.893 |  | 0.091 | 0.190 | 0.536 | 0.757 |
| *Sutterellaceae* | 0.075 | 0.121 | 0.603 | 0.851 |  | 0.080 | 0.063 | 0.115 | 0.394 |
| *Acidaminococcaceae* | 0.001 | 0.035 | 0.155 | 0.632 |  | 0.024 | 0.196 | 0.070 | 0.300 |
| *Actinomycetaceae* | 0.045 | 0.051 | 0.590 | 0.851 |  | 0.090 | 0.077 | 0.229 | 0.600 |
| *Clostridiales IS XI* | 0.592 | 0.013 | 0.368 | 0.632 |  | 0.032 | 0.026 | 0.377 | 0.675 |
| *Eubacteriaceae* | 0.003 | 0.052 | 0.372 | 0.632 |  | 0.029 | 0.047 | 0.655 | 0.827 |
| *Fusobacteriaceae* | 0.007 | 0.026 | 0.883 | 0.921 |  | 0.016 | 0.025 | 0.275 | 0.600 |
| *Carnobacteriaceae* | 0.023 | 0.017 | 0.274 | 0.632 |  | 0.028 | 0.022 | 0.394 | 0.675 |

a) p-value of unpaired T-test (Alpha diversity measures) or Mann-Whitney test (Family level relative abundance) between infants born by C-section compared to vaginal birth.

b) q-value indicates False Discovery Rate corrected p-values. Significant p- and q-values are in bold.
