# Supplementary material for: Infant Gut Microbiota Development Is Driven by Transition to Family Foods Independent of Maternal Obesity
Source: mSphere. 2016 Feb 10;1(1):e00069-15. doi: 10.1128/mSphere.00069-15 (PMC4863607; doi:10.1128/mSphere.00069-15)
Supplement: Table S3 [file sph001162013st5.docx]

|  | **SKOT I** | |  | **SKOT II** | |
| --- | --- | --- | --- | --- | --- |
| **Gestational age at birth (mean ± sd)** | 40.2 ± 1.2 | |  | 40.3 ± 1.3 | |
|  | **Pearson’s correlations** | | | | |
|  | **r** | **p-value^a^** |  | **r** | **p-value^a^** |
| **Alpha diversity** |  |  |  |  |  |
| Shannon index | 0.065 | 0.512 |  | -0.051 | 0.619 |
| Observed genera | 0.079 | 0.420 |  | -0.083 | 0.423 |
| Pielou’s evenness index | 0.043 | 0.666 |  | -0.040 | 0.699 |
| **Family level relative abundance** |  |  |  |  |  |
| *Lachnospiraceae* | -0.002 | 0.980 |  | -0.021 | 0.832 |
| *Bifidobacteriaceae* | 0.065 | 0.504 |  | -0.056 | 0.580 |
| *Bacteroidaceae* | 0.002 | 0.981 |  | -0.114 | 0.255 |
| *Ruminococcaceae* | 0.020 | 0.836 |  | -0.034 | 0.739 |
| *Veillonellaceae* | 0.079 | 0.412 |  | 0.069 | 0.495 |
| *Enterobacteriaceae* | -0.165 | 0.086 |  | 0.158 | 0.114 |
| *Coriobacteriaceae* | 0.031 | 0.751 |  | 0.031 | 0.759 |
| *Erysipelotrichaceae* | -0.004 | 0.970 |  | 0.003 | 0.974 |
| *Streptococcaceae* | -0.045 | 0.644 |  | -0.040 | 0.690 |
| *Peptostreptococcaceae* | -0.041 | 0.671 |  | 0.021 | 0.835 |
| *Clostridiaceae* | -0.030 | 0.757 |  | -0.058 | 0.563 |
| *Prevotellaceae* | -0.059 | 0.543 |  | -0.131 | 0.193 |
| *Enterococcaceae* | 0.108 | 0.263 |  | 0.033 | 0.741 |
| *Lactobacillaceae* | 0.050 | 0.608 |  | -0.054 | 0.595 |
| *Porphyromonadaceae* | -0.052 | 0.591 |  | 0.116 | 0.246 |
| *Rikenellaceae* | -0.074 | 0.447 |  | 0.031 | 0.757 |
| *Pasteurellaceae* | -0.036 | 0.712 |  | -0.132 | 0.187 |
| *Sutterellaceae* | 0.011 | 0.907 |  | -0.067 | 0.509 |
| *Acidaminococcaceae* | 0.072 | 0.454 |  | -0.012 | 0.904 |
| *Actinomycetaceae* | -0.114 | 0.236 |  | -0.030 | 0.765 |
| *Clostridiales IS XI* | -0.068 | 0.482 |  | -0.029 | 0.772 |
| *Eubacteriaceae* | 0.025 | 0.799 |  | 0.050 | 0.619 |
| *Fusobacteriaceae* | -0.024 | 0.804 |  | 0.087 | 0.389 |
| *Carnobacteriaceae* | 0.025 | 0.799 |  | -0.098 | 0.329 |

a) p-value of Pearson’s correlations between gestational age at birth and alpha diversity measures and family level composition of gut microbiota.
