# Supplementary material for: Infant Gut Microbiota Development Is Driven by Transition to Family Foods Independent of Maternal Obesity
Source: mSphere. 2016 Feb 10;1(1):e00069-15. doi: 10.1128/mSphere.00069-15 (PMC4863607; doi:10.1128/mSphere.00069-15)
Supplement: Table S4 [file sph001162013st6.docx]

| **SKOT I+II** |  | **Oral antibiotics** | **No antibiotics** | **p-value^a^** |
| --- | --- | --- | --- | --- |
| **9 months** | **Number of infants** | **n = 3** | **n = 224** | - |
|  | Shannon index (mean ± sd) | 1.53 ± 0.37 | 1.75 ± 0.40 | 0.333 |
|  | Observed genera (mean ± sd) | 29.42 ± 1.28 | 33.67 ± 6.74 | 0.276 |
|  | Pielou’s evenness index (mean ± sd) | 0.45 ± 0.11 | 0.50 ± 0.10 | 0.431 |
| **18 months** | **Number of infants** | **n = 7** | **n = 220** | - |
|  | Shannon index (mean ± sd) | 2.01 ± 0.35 | 2.11 ± 0.31 | 0.445 |
|  | Observed genera (mean ± sd) | 37.93 ± 9.33 | 39.81 ± 5.87 | 0.415 |
|  | Pielou’s evenness index (mean ± sd) | 0.56 ± 0.07 | 0.57 ± 0.07 | 0.555 |

a) p-value of unpaired t-test.
