# Supplementary material for: Infant Gut Microbiota Development Is Driven by Transition to Family Foods Independent of Maternal Obesity
Source: mSphere. 2016 Feb 10;1(1):e00069-15. doi: 10.1128/mSphere.00069-15 (PMC4863607; doi:10.1128/mSphere.00069-15)
Supplement: Table S5 [file sph001162013st7.docx]

| **Food Group [g/day/kg]** | ***Lachnospiraceae*** | ***Bifidobacteriaceae*** | ***Bacteroidaceae*** | ***Ruminococcaceae*** | ***Veillonellaceae*** | ***Enterobacteriaceae*** | ***Coriobacteriaceae*** | ***Erysipelotrichaceae*** | ***Streptococcaceae*** | ***Peptostreptococcaceae*** | ***Clostridiaceae*** | ***Prevotellaceae*** | ***Enterococcaceae*** | ***Lactobacillaceae*** | ***Porphyromonadaceae*** | ***Rikenellaceae*** | ***Pasteurellaceae*** | ***Sutterellaceae*** | ***Acidaminococcaceae*** | ***Actinomycetaceae*** | ***Clostridiales***  ***Incertae Sedis XI*** | ***Eubacteriaceae*** | ***Fusobacteriaceae*** | ***Carnobacteriaceae*** |
| --- | --- | --- | --- | --- | --- | --- | --- | --- | --- | --- | --- | --- | --- | --- | --- | --- | --- | --- | --- | --- | --- | --- | --- | --- |
| **Porridge** | **0.086** | **0.086** | 0.125 | 0.467 | 0.477 | 0.972 | 0.945 | 0.820 | 0.704 | **0.011** | 0.467 | 0.988 | 0.936 | 0.411 | 0.832 | 0.970 | 0.065 | 0.986 | 0.832 | 0.411 | 0.467 | 0.704 | 0.186 | 0.767 |
| **BreakfastCereals** | 0.991 | 0.991 | 0.954 | 0.886 | 0.991 | 0.991 | 0.702 | 0.991 | 0.991 | 0.690 | 0.991 | 0.702 | 0.690 | 0.690 | 0.991 | 0.991 | 0.690 | 0.690 | 0.690 | 0.991 | 0.690 | 0.690 | 0.316 | 0.690 |
| **WheatBreadWholegrain** | 0.840 | 0.706 | 0.706 | 0.706 | 0.706 | 0.782 | 0.550 | 0.706 | 0.706 | 0.983 | 0.782 | 0.840 | 0.550 | 0.706 | 0.787 | 0.787 | 0.787 | 0.752 | 0.782 | 0.706 | 0.782 | 0.840 | 0.706 | 0.745 |
| **WheatBreadNoWholegrain** | 0.151 | 0.437 | 0.514 | 0.455 | 0.151 | 0.239 | 0.831 | 0.169 | 0.158 | 0.514 | 0.514 | 0.158 | 0.396 | 0.948 | 0.791 | 0.948 | 0.868 | 0.127 | 0.396 | 0.514 | 0.127 | 0.232 | 0.940 | 0.396 |
| **RyeBread** | 0.546 | 0.589 | 0.677 | 0.219 | 0.589 | 0.191 | 0.585 | 0.663 | 0.585 | 0.596 | 0.596 | 0.663 | 0.129 | 0.585 | 0.589 | 0.589 | 0.546 | 0.219 | 0.589 | 0.585 | 0.420 | 0.446 | 0.596 | 0.821 |
| **PastaRice** | 0.589 | 0.619 | 0.979 | 0.589 | 0.979 | 0.589 | 0.956 | 0.924 | 0.619 | 0.956 | 0.619 | 0.924 | 0.589 | 0.589 | 0.956 | 0.956 | 0.648 | 0.589 | 0.979 | 0.956 | 0.589 | 0.619 | 0.924 | 0.589 |
| **Potato** | 0.922 | 0.436 | 0.469 | 0.373 | 0.922 | 0.940 | 0.373 | 0.213 | 0.922 | 0.940 | 0.940 | 0.327 | 0.373 | 0.469 | 0.469 | 0.922 | **0.004** | 0.940 | 0.940 | **0.028** | 0.109 | 0.940 | 0.156 | 0.940 |
| **Fruit** | 0.969 | 0.462 | 0.969 | 0.969 | 0.969 | 0.969 | 0.969 | 0.969 | 0.969 | 0.902 | 0.969 | 0.969 | 0.969 | 0.969 | 0.969 | 0.969 | 0.153 | 0.969 | 0.969 | 0.192 | 0.462 | 0.969 | 0.969 | 0.969 |
| **Vegetable** | 0.388 | **0.072** | 0.583 | 0.307 | 0.713 | 0.850 | 0.991 | 0.572 | 0.583 | 0.572 | 0.572 | 0.439 | 0.432 | 0.583 | 0.575 | 0.479 | **0.072** | 0.721 | 0.713 | 0.148 | 0.148 | 0.295 | 0.572 | 0.703 |
| **Fish** | 0.803 | 0.289 | 0.317 | 0.797 | 0.526 | 0.929 | 0.317 | 0.762 | 0.414 | 0.317 | 0.526 | 0.317 | 0.421 | 0.317 | 0.289 | 0.728 | **0.003** | 0.732 | 0.289 | 0.289 | 0.317 | 0.929 | 0.289 | 0.929 |
| **Meat** | **0.085** | **0.046** | 0.440 | **0.073** | 0.451 | 0.423 | 0.614 | 0.898 | 0.614 | 0.614 | 0.614 | 0.142 | **0.054** | 0.629 | 0.430 | 0.819 | 0.430 | **0.019** | 0.986 | 0.252 | 0.430 | **0.084** | 0.614 | 0.959 |
| **Poultry** | 0.768 | 0.768 | 0.768 | 0.768 | 0.768 | 0.768 | 0.768 | 0.768 | 0.768 | 0.272 | 0.768 | 0.768 | 0.768 | 0.768 | 0.953 | 0.986 | 0.768 | 0.768 | 0.768 | 0.768 | 0.272 | 0.768 | 0.953 | 0.309 |
| **Egg** | 0.785 | 0.524 | 0.879 | 0.818 | 0.818 | 0.804 | 0.804 | 0.524 | 0.785 | 0.818 | 0.851 | 0.851 | 0.512 | 0.818 | 0.865 | 0.818 | 0.347 | 0.126 | 0.902 | 0.851 | 0.347 | 0.818 | 0.818 | 0.851 |
| **FatsAnimal** | 0.731 | 0.862 | 0.690 | 0.862 | 0.838 | 0.731 | 0.862 | 0.791 | 0.838 | 0.731 | 0.731 | 0.838 | 0.731 | 0.883 | 0.690 | 0.879 | 0.690 | 0.731 | 0.838 | 0.731 | 0.731 | 0.862 | 0.731 | 0.838 |
| **FatsVegetable** | 0.844 | 0.622 | 0.844 | 0.844 | 0.844 | 0.844 | 0.934 | 0.934 | 0.966 | 0.844 | 0.844 | 0.934 | 0.844 | 0.923 | 0.844 | 0.844 | 0.622 | 0.844 | 0.844 | 0.844 | 0.582 | 0.934 | 0.844 | 0.844 |
| **Cheese** | 0.172 | 0.121 | 0.946 | 0.946 | 0.647 | 0.946 | 0.515 | 0.515 | 0.623 | 0.172 | 0.621 | 0.946 | 0.812 | 0.946 | 0.946 | 0.946 | 0.946 | 0.433 | 0.812 | 0.946 | 0.946 | 0.946 | 0.946 | 0.946 |
| **Milk** | 0.297 | 0.666 | 0.894 | 0.737 | 0.736 | 0.666 | 0.756 | 0.666 | 0.264 | **0.058** | 0.207 | 0.207 | 0.970 | 0.666 | 0.970 | 0.704 | 0.736 | 0.894 | 0.876 | 0.247 | 0.876 | 0.736 | 0.666 | **0.058** |
| **Formula** | **0.003** | 0.376 | 0.376 | 0.812 | **0.003** | **0.079** | 0.251 | **0.003** | 0.376 | 0.376 | 0.056 | 0.132 | 0.600 | **0.015** | 0.376 | 0.465 | **0.056** | 0.376 | 0.376 | 0.376 | 0.851 | 0.251 | 0.189 | 0.437 |
| **BreastMilk** | **<0.0001** | **<0.0001** | 0.491 | 0.133 | **<0.0001** | **0.016** | 0.698 | **0.001** | 0.586 | **<0.0001** | 0.715 | 0.669 | **0.007** | **0.026** | 0.116 | 0.847 | 0.139 | 0.678 | 0.669 | 0.421 | 0.727 | **0.018** | **0.090** | 0.853 |
| **FruitNutSnack** | 0.902 | 0.880 | 0.828 | 0.861 | 0.828 | 0.828 | 0.828 | 0.902 | 0.880 | 0.828 | 0.880 | 0.828 | 0.902 | 0.880 | 0.861 | 0.828 | 0.880 | 0.828 | 0.828 | 0.861 | 0.828 | 0.828 | 0.880 | 0.880 |
| **SweetsCake** | 0.706 | 0.361 | 0.671 | 0.238 | 0.238 | **0.024** | 0.361 | 0.671 | 0.274 | 0.274 | 0.381 | 0.540 | **0.063** | 0.361 | 0.361 | 0.361 | 0.805 | 0.225 | 0.690 | 0.671 | 0.361 | 0.706 | 0.228 | 0.381 |
| **SugaryDrink** | 0.696 | 0.525 | 0.775 | 0.367 | 0.775 | 0.775 | 0.339 | 0.775 | 0.972 | 0.972 | 0.972 | 0.972 | 0.367 | 0.972 | 0.148 | **0.089** | 0.972 | 0.148 | 0.446 | 0.367 | 0.773 | 0.446 | 0.173 | 0.266 |
| **FastFood** | 0.812 | 0.812 | 0.967 | 0.170 | 0.840 | 0.170 | 0.840 | 0.413 | 0.840 | 0.812 | 0.840 | 0.812 | 0.413 | 0.812 | 0.812 | 0.812 | 0.812 | 0.413 | 0.812 | 0.762 | 0.812 | 0.762 | 0.812 | 0.812 |
| **PC1 (Family Food)** | **0.009** | **0.008** | 0.522 | 0.117 | 0.318 | 0.175 | 0.908 | 0.979 | 0.588 | 0.841 | 0.340 | 0.417 | **0.019** | 0.841 | 0.359 | 0.511 | **0.030** | **0.009** | 0.869 | 0.166 | 0.117 | 0.222 | 0.431 | 0.511 |
| **PC2 (Health-Conscious)** | 0.195 | **0.021** | 0.195 | 0.548 | 0.668 | 0.664 | 0.839 | 0.401 | 0.488 | 0.668 | 0.717 | 0.735 | 0.668 | 0.195 | 0.839 | 0.668 | **0.012** | 0.668 | 0.839 | **0.031** | **0.013** | 0.668 | 0.195 | 0.668 |
