# Supplementary material for: Infant Gut Microbiota Development Is Driven by Transition to Family Foods Independent of Maternal Obesity
Source: mSphere. 2016 Feb 10;1(1):e00069-15. doi: 10.1128/mSphere.00069-15 (PMC4863607; doi:10.1128/mSphere.00069-15)
Supplement: Table S6 [file sph001162013st8.docx]

| **Family level relative abundance** | **Duration exclusively breastfeeding (0-6 months)** | | | |  | **Total duration breastfeeding (0-18 months)** | | | |
| --- | --- | --- | --- | --- | --- | --- | --- | --- | --- |
|  | **SKOT I** | | **SKOT II** | |  | **SKOT I** | | **SKOT II** | |
|  | **rho** | **p-value^a^** | **rho** | **p-value^a^** |  | **rho** | **p-value^a^** | **rho** | **p-value^a^** |
| *Lachnospiraceae* | -0.056 | 0.556 | -0.119 | 0.217 |  | -0.035 | 0.725 | -0.058 | 0.556 |
| *Bifidobacteriaceae* | -0.200 | **0.033** | -0.082 | 0.396 |  | -0.062 | 0.532 | -0.026 | 0.791 |
| *Bacteroidaceae* | 0.140 | 0.137 | -0.013 | 0.892 |  | 0.105 | 0.291 | -0.014 | 0.889 |
| *Ruminococcaceae* | 0.153 | 0.104 | 0.159 | 0.099 |  | 0.168 | 0.090 | 0.074 | 0.451 |
| *Veillonellaceae* | 0.088 | 0.352 | -0.083 | 0.389 |  | 0.019 | 0.847 | -0.034 | 0.731 |
| *Enterobacteriaceae* | -0.141 | 0.134 | -0.242 | **0.011** |  | -0.031 | 0.755 | -0.210 | **0.031** |
| *Coriobacteriaceae* | -0.229 | **0.014** | 0.026 | 0.788 |  | -0.239 | **0.015** | -0.019 | 0.847 |
| *Erysipelotrichaceae* | 0.035 | 0.711 | 0.078 | 0.420 |  | -0.145 | 0.143 | 0.063 | 0.519 |
| *Streptococcaceae* | -0.056 | 0.556 | -0.115 | 0.235 |  | 0.049 | 0.624 | 0.008 | 0.937 |
| *Peptostreptococcaceae* | -0.109 | 0.250 | -0.009 | 0.928 |  | -0.180 | 0.069 | 0.017 | 0.863 |
| *Clostridiaceae* | -0.089 | 0.348 | -0.100 | 0.299 |  | -0.260 | **0.008** | -0.050 | 0.613 |
| *Prevotellaceae* | 0.065 | 0.492 | 0.113 | 0.240 |  | 0.038 | 0.705 | 0.134 | 0.170 |
| *Enterococcaceae* | 0.069 | 0.465 | -0.120 | 0.214 |  | 0.161 | 0.105 | 0.040 | 0.687 |
| *Lactobacillaceae* | -0.107 | 0.258 | 0.007 | 0.944 |  | -0.003 | 0.973 | 0.098 | 0.319 |
| *Porphyromonadaceae* | -0.138 | 0.142 | 0.024 | 0.807 |  | -0.058 | 0.562 | -0.104 | 0.287 |
| *Rikenellaceae* | 0.114 | 0.226 | -0.029 | 0.764 |  | 0.145 | 0.144 | -0.002 | 0.984 |
| *Pasteurellaceae* | -0.037 | 0.698 | -0.092 | 0.342 |  | -0.073 | 0.463 | 0.046 | 0.640 |
| *Sutterellaceae* | 0.078 | 0.412 | 0.048 | 0.617 |  | 0.055 | 0.582 | 0.148 | 0.130 |
| *Acidaminococcaceae* | -0.054 | 0.568 | 0.082 | 0.394 |  | 0.003 | 0.977 | -0.018 | 0.856 |
| *Actinomycetaceae* | 0.002 | 0.983 | -0.168 | 0.081 |  | -0.032 | 0.745 | -0.025 | 0.800 |
| *Clostridiales Incertae Sedis XI* | -0.095 | 0.314 | -0.070 | 0.468 |  | -0.077 | 0.442 | -0.029 | 0.769 |
| *Eubacteriaceae* | -0.051 | 0.586 | -0.172 | 0.074 |  | 0.043 | 0.665 | -0.104 | 0.288 |
| *Fusobacteriaceae* | 0.135 | 0.151 | -0.016 | 0.872 |  | 0.103 | 0.301 | 0.074 | 0.449 |
| *Carnobacteriaceae* | 0.094 | 0.317 | -0.172 | 0.073 |  | 0.084 | 0.400 | 0.088 | 0.368 |

a) p-value of Spearman’s rank correlation between duration of exclusive/total breastfeeding and family level composition of gut microbiota. Significant p-values are in bold. None were significant after False Discovery Rate correction of p-values.
