# Supplementary material for: Infant Gut Microbiota Development Is Driven by Transition to Family Foods Independent of Maternal Obesity
Source: mSphere. 2016 Feb 10;1(1):e00069-15. doi: 10.1128/mSphere.00069-15 (PMC4863607; doi:10.1128/mSphere.00069-15)
Supplement: Table S7 [file sph001162013st9.docx]

| **Family level relative abundance** | **Age at introduction of complementary feeding (3-6 months)** | | | | | |
| --- | --- | --- | --- | --- | --- | --- |
|  | **SKOT I** | | | **SKOT II** | | |
|  | **rho** | **p-value^a^** | **q-value** | **rho** | **p-value^a^** | **q-value** |
| *Lachnospiraceae* | -0.123 | 0.194 | Øverst på formularen  0.644Nederst på formularen | -0.187 | **0.047** | Øverst på formularen  0.378Nederst på formularen |
| *Bifidobacteriaceae* | 0.070 | 0.461 | 0.790 | 0.103 | 0.275 | 0.826 |
| *Bacteroidaceae* | -0.034 | 0.720 | 0.925 | -0.011 | 0.905 | 0.945 |
| *Ruminococcaceae* | 0.076 | 0.420 | 0.776 | -0.046 | 0.630 | 0.913 |
| *Veillonellaceae* | 0.150 | 0.111 | 0.507 | 0.128 | 0.176 | 0.826 |
| *Enterobacteriaceae* | 0.088 | 0.350 | 0.699 | 0.118 | 0.212 | 0.826 |
| *Coriobacteriaceae* | -0.152 | 0.106 | 0.507 | -0.084 | 0.379 | 0.841 |
| *Erysipelotrichaceae* | -0.144 | 0.127 | 0.507 | 0.030 | 0.755 | 0.913 |
| *Streptococcaceae* | 0.064 | 0.498 | 0.797 | 0.078 | 0.411 | 0.841 |
| *Peptostreptococcaceae* | -0.150 | 0.112 | 0.507 | -0.005 | 0.959 | 0.959 |
| *Clostridiaceae* | 0.099 | 0.296 | 0.699 | 0.106 | 0.265 | 0.826 |
| *Prevotellaceae* | 0.011 | 0.905 | 0.974 | 0.240 | **0.010** | 0.125 |
| *Enterococcaceae* | -0.027 | 0.776 | 0.931 | 0.084 | 0.376 | 0.841 |
| *Lactobacillaceae* | -0.032 | 0.732 | 0.925 | 0.042 | 0.657 | 0.913 |
| *Porphyromonadaceae* | -0.149 | 0.114 | 0.507 | -0.014 | 0.882 | 0.945 |
| *Rikenellaceae* | 0.117 | 0.215 | 0.644 | -0.058 | 0.538 | 0.913 |
| *Pasteurellaceae* | 0.108 | 0.252 | 0.671 | 0.259 | **0.006** | 0.125 |
| *Sutterellaceae* | -0.017 | 0.860 | 0.974 | -0.029 | 0.761 | 0.913 |
| *Acidaminococcaceae* | -0.052 | 0.582 | 0.874 | -0.072 | 0.447 | 0.841 |
| *Actinomycetaceae* | -0.003 | 0.976 | 0.976 | 0.071 | 0.455 | 0.841 |
| *Clostridiales Incertae Sedis XI* | -0.092 | 0.330 | 0.699 | 0.110 | 0.246 | 0.826 |
| *Eubacteriaceae* | -0.173 | 0.065 | 0.507 | -0.051 | 0.594 | 0.913 |
| *Fusobacteriaceae* | -0.008 | 0.934 | 0.974 | 0.032 | 0.733 | 0.913 |
| *Carnobacteriaceae* | -0.033 | 0.730 | 0.925 | -0.017 | 0.861 | 0.945 |

a) p-value of Spearman’s rank correlation between age at introduction of complementary feeding and family level composition of gut microbiota. Significant p-values are in bold. None were significant after False Discovery Rate correction of p-values.
